# Cystadenocarcinoma of the gallbladder: A case report

**DOI:** 10.1016/j.amsu.2022.104955

**Published:** 2022-11-17

**Authors:** Y. Hammami, A. Sebai, Y. Ouadi, A. Ben Mahmoud, W. Frikha, J. Bel Hadj Ali, S. Fteriche, J.M. Kacem

**Affiliations:** a Department of surgery, Rabta Hospital, Tunisia; b Department of Radiology, Rabta Hospital, Tunisia

**Keywords:** Case report, Cystadenocarcinoma, Gallbladder, Bisegmentectomy, General surgery

## Abstract

**Introduction and importance:**

Cystadenoma and cystadenocarcinoma of the biliary duct remain a rare diagnosis, the localization is intrahepatic in 97% of the cases and extrahepatic in 3%of them [1]. Cystadenocarcinoma of the gallbladder is the rarest localization, it's usually diagnosed late because of the poor symptoms and the nonspecific lesions in the different imaging.

Only hundreds of cases have been reported for cystadenocarcinoma of the gallbladder.

This case report has been reported in line with the SCARE Criteria.

**Case presentation:**

We present a 70-year-old woman, with no family or personal history, who was complaining of pain in the right hypochondrium and an altered general well-being, with no other clinical abnormalities.

The imaging including abdominal sonography, CT scan, and MRI concluded at a budding lesion formed in the gallbladder wall which measured 65 × 15mm.

Cancer of the gallbladder was suspected and the patient was operated. A resection of segment s4b and 5 of the liver was performed with lymphadenectomy. The post-operative clinical course was uneventful.

The diagnosis of cystadenocarcinoma of the gallbladder was confirmed on an anatomopathological exam of the specimen.

**Conclusion:**

Cystadenocarcinoma is a rare diagnosis.

There are no specific symptoms or lesions at the imaging.

The confirmation is obtained on an anatomopathological study of the specimen.

## Highlights

• Cystadenocarcinoma is a rare diagnosis.
• There are no specific symptoms nor lesions at the imaging.
• The confirmation is done on an anatomopathological study on the specimen.

## 1 Introduction

Cystadenoma and cystadenocarcinoma of the biliary ducts are rare tumors. The first case to be reported was by EDMONSON in 1958 [1].

It is usually observed in the intra or extrahepatic bile ducts [1], and the gallbladder is the rarest localization of this carcinoma. Differential diagnosis like parasitic cysts, chronic cholecystitis, or abscess of liver, make the pattern recognition of the cystadenocarcinoma difficult.

## 2 Case presentation

A 70-year-old woman, with no family or personal history, was complaining of pain in the right hypochondrium, and an altered general well-being with no other symptoms.

Examination showed tenderness in the right hypochondrium, with no jaundice and no palpable abdominal mass.

Hepatic blood tests were normal. Carcinoembryonic antigen (CEA) and Carbohydrate Antigen (CA19-9) were normal. Alpha-fetoprotein (AFP) was above the superior limit.

The abdominal Sonography showed a thick-walled hyperechoic lesion with lithiasis of the gallbladder.

**Fig. 1:**
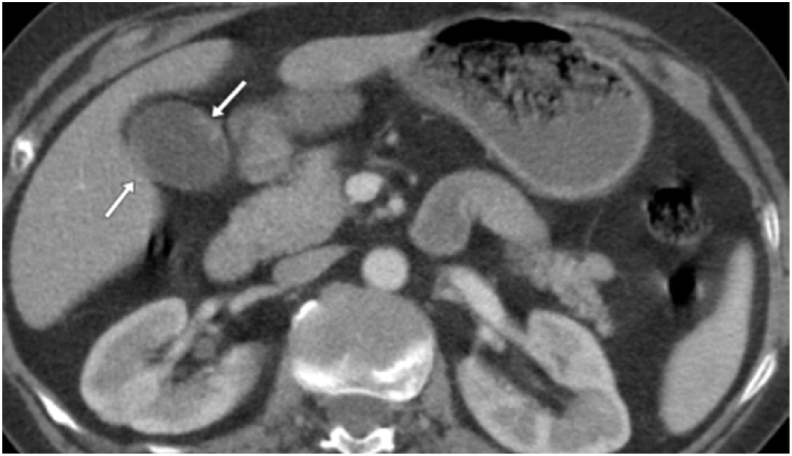
Arterial sequence of the CT scan.

The CT scan showed a bifocal hypodense lesion in the gallbladder wall (Fig. 1)

**Fig. 2:**
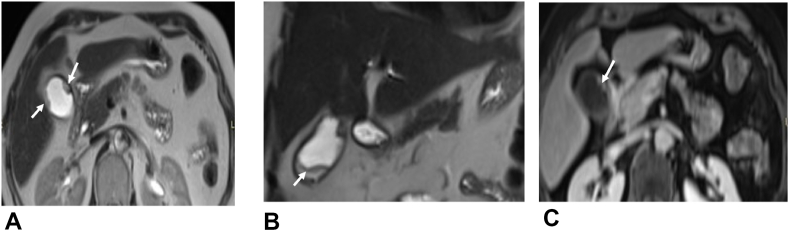
Bifocal parietal thickening (arrows) of the body of the gallbladder in hyposignal T2, strongly enhanced after contrast, without restriction of diffusion. a) axial sequence T SS FSE b) diffusion sequence b 800 c) T EG fat sat Gado portal sequence.

The MRI showed a bi-focal lesion developed on the wall of the gallbladder that measured 65 × 15mm associated with multiple lithiasis (Fig. 2).

A cancer of the gallbladder was suspected and we opted for a surgical treatment.

**Fig. 3:**
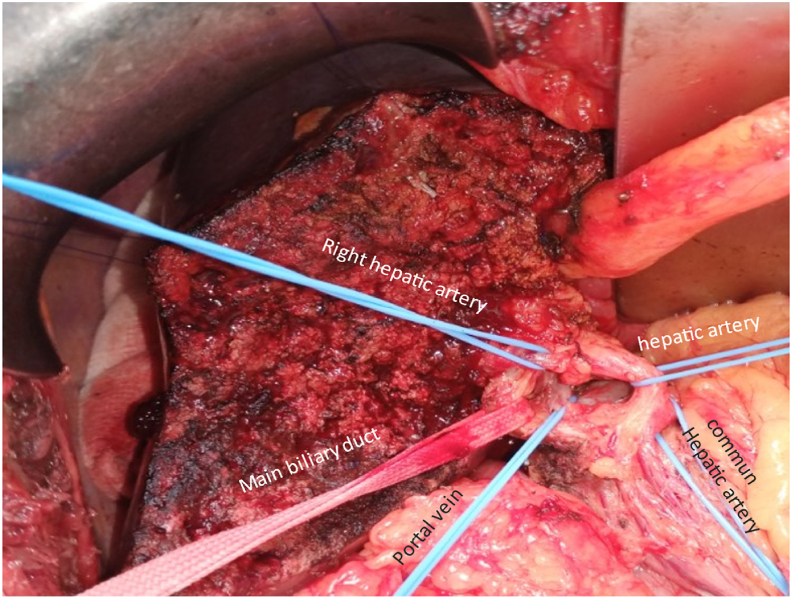
Bisegmentectomy IVb-V with extensive lymph node dissection.

A bi-segmentectomy s4b and s5 of the liver with extended lymphadenectomy were performed by a professor in general surgery in la Rabta hospital in Tunisia (Fig. 3).

The diagnosis of cystadenocarcinoma of the gallbladder was confirmed on an anatomopathological exam of the specimen classified as pT2aN0 with harvested lymph nodes of 15, all being reactive.

The post-operative clinical course was uneventful. The patient noted an improvement in her general wellbeing.

The follow-up based on multiple CT scans and tumor markers was regularly conducted until august 2022. It showed no recurrence of the tumor.

## 3 Clinical discussion

Cystadenocarcinoma of the biliary duct is usually observed in women, at the age range of 40–60 years Old. In a histopathologic study of 540 consecutive cholecystectomies, Terada [2] reported a rate of 0.20% of cystadenocarcinoma.

It has no specific symptoms. The most common complaint by the patients is right hypochondoralgia or bloating.

Examination usually shows tenderness in the right hypochondrium, sometimes a palpable mass in the upper abdomen or even jaundice.

CA19-9 and ACE are not specific for cystadenocarcinoma. Sang [3] reported, based on the data of the comparison study including 33 cases of cystadenoma and cystadenocarcinoma, that levels of CA19-9 are not more specific for cystadenocarcinoma than cystadenoma.

Ultrasonography is the first imaging indicated. Lesions may vary and are not specific for cystadenocarcinoma. We essentially observe a thick-walled lesion developed in the gallbladder as observed in our case. Sometimes we find a cystic lesion with many septations and lymph nodes.

The MRI is more specific to define benign and malignant lesions [3].

A fine needle aspiration for cytological study must be avoided to prevent the spreading of malignant cells [4]. The confirmation of the diagnosis is made on an anatomopathological examination of a specimen.

As a malignant lesion is suspected, we perform a radical surgery based on a liver resection s4b and 5 with extensive lymphadenectomy.

Even though the prognosis of our patient is prosperous and the surgical treatment was radical, recurrence may be occurred in 15% of the cases [5].

A regular follow up with clinical examination, tumor markers and CT scan for 5 years is advised.

## 4 Conclusion

Cystadenocarcinoma of the biliary duct is rare, mostly intrahepatic.

The extra hepatic lesions including the gallbladder, like the one we represent in our case report represent 3% of all cases combined.

The symptoms are poor and the tumor markers aren't specific. The lesions at the imaging only show thickening off the wall, or a cystic lesion with multiples septations.

Bisegmentectmy of the liver s4b and 5 with lymph node resection is a complete and sufficient treatment.

The diagnosis of cystadenocarcinoma of the liver is made on an anatomopathological exam of the specimen.

Locoregional or distant recurrence is observed in 15% of the cases even after complete resection with margin-negative surgery.

## Ethical approval

Not applicable.

## Sources of funding

No sources of funding.

## Author contribution

Hammami yasmine, Conceptualisation, Redaction, Data curation, Project administration/Sebai amine, Conceptualisation, Redaction, Data curation, Project administration/Ouadi Yacine Conceptualisation, Redaction,/Ben Mahmoud Ahmed Photography rendering/Frikha Wassim Photography rendering, Data curation/Bel haj Ali jihene Data curation/Fterich Fadhel Samir Supervision, Validation, Visualisation/Montasser Kacem Supervision, Validation, Visualisation.

## Registration of research studies

Name of the registry:

Unique Identifying number or registration ID:

Hyperlink to your specific registration (must be publicly accessible and will be checked):

## Guarantor

Hammami Yasmine.

## Consent

Written informed consent was obtained from the patient for publication of this case report and any accompanying images. A copy of the written consent is available for review by the Editor-in-Chief of this journal on request.

## Research registration

Not applicable.

## Provenance and per review

Not commissioned, externally pee-reviewed.

## Declaration of competing interest

All authors declare that they have no any conflicts of interest.
